# Surveillance of health care associated infections in bavarian intensive care units

**DOI:** 10.1186/2047-2994-4-S1-P238

**Published:** 2015-06-16

**Authors:** C Höller, N Grundmann, U Kandler, S Kolb, S Nickel, E Tomasic, G Valenza, V Lehner-Reindl

**Affiliations:** 1 Bavarian Health and Food Safety Authority, Germany

## Introduction

Surveillance of health care associated infections (HAI) has been mandatory in Germany since the year 2001.

## Objectives

The Bavarian Health and Food Safety Authority (LGL) aimed to assess whether this legal demand was met by the hospitals and if the results of surveillance induces changes concerning management of hospital hygiene.

## Methods

Prior to a Bavarian wide monitoring program performed by the public health offices the special unit for hospital hygiene (SEI) of the LGL developed a checklist in order to facilitate a standardized monitoring. This checklist was evaluated by the SEI in a pilot study comprising intensive care units (ICU) of 40 hospitals. In the following year 284 ICUs of 395 Bavarian hospitals were monitored by the public health offices. The checklists were returned to the LGL and were analyzed by the SEI.

## Results

75 % of hospitals stated that they perform HAI surveillance in their ICUs, 19 % answered the question in the negative and 6 % of data were missing. The surveillance system KISS provided by the National Reference Institute was used in 33% of the ICUs, in 35 % an in-house method based on KISS and in 18 % some other kind of method was used. Some hospitals had implemented several methods for surveillance. ICUs using other methods than KISS did not have written standards to define the specific HAI they were looking for or a denominator in almost a third of the cases. The data for surveillance were collected and analyzed predominantly by the infection control staff. However, there seems to exist a communication gap. The data are not shared with the head of the ICU in 15 %, the ward doctors in 23 % and the nursing staff in 35 %. The quality management is not informed in 53 % and the data are published rather seldom in the official quality reports of the hospitals (15 %). Not only the communication of data could and should be improved but also the understanding why these data are collected in the first place. Nearly all ICUs answered that they would react on data in general, but 16% did nothing specifically.

## Conclusion

In summary the monitoring program showed that most hospitals performed some method of surveillance for HAIs in their ICUS, but that partly deficiencies which would be fairly easy to overcome are still in place.

## Disclosure of interest

None declared.

